# An integrated RF-receive/B_0_-shim array coil boosts performance of whole-brain MR spectroscopic imaging at 7 T

**DOI:** 10.1038/s41598-020-71623-5

**Published:** 2020-09-14

**Authors:** Morteza Esmaeili, Jason Stockmann, Bernhard Strasser, Nicolas Arango, Bijaya Thapa, Zhe Wang, Andre van der Kouwe, Jorg Dietrich, Daniel P. Cahill, Tracy T. Batchelor, Jacob White, Elfar Adalsteinsson, Lawrence Wald, Ovidiu C. Andronesi

**Affiliations:** 1 Athinoula A. Martinos Center for Biomedical Imaging, Department of Radiology, Massachusetts General Hospital, Harvard Medical School, Boston, MA USA; 2 Department of Diagnostic Imaging, Akershus University Hospital, Lørenskog, Norway; 3 Department of Electrical Engineering and Computer Science, Massachusetts Institute of Technology, Cambridge, MA USA; 4 Siemens Medical Solutions, USA, Charlestown, MA USA; 5 Division of Neuro-Oncology, Department Neurology, Massachusetts General Hospital, Harvard Medical School, Boston, MA USA; 6 Department of Neurosurgery, Massachusetts General Hospital, Harvard Medical School, Boston, MA USA; 7 Department Neurology, Brigham’s and Women Hospital, Harvard Medical School, Boston, MA USA; 8 Athinoula A. Martinos Center for Biomedical Imaging, Building 149, Room 2301 13th Street, Charlestown, MA 02129 USA

**Keywords:** Biomedical engineering, Cancer imaging, Cancer metabolism, Imaging techniques, Neurochemistry

## Abstract

Metabolic imaging of the human brain by in-vivo magnetic resonance spectroscopic imaging (MRSI) can non-invasively probe neurochemistry in healthy and disease conditions. MRSI at ultra-high field (≥ 7 T) provides increased sensitivity for fast high-resolution metabolic imaging, but comes with technical challenges due to non-uniform B_0_ field. Here, we show that an integrated RF-receive/B_0_-shim (AC/DC) array coil can be used to mitigate 7 T B_0_ inhomogeneity, which improves spectral quality and metabolite quantification over a whole-brain slab. Our results from simulations, phantoms, healthy and brain tumor human subjects indicate improvements of global B_0_ homogeneity by 55%, narrower spectral linewidth by 29%, higher signal-to-noise ratio by 31%, more precise metabolite quantification by 22%, and an increase by 21% of the brain volume that can be reliably analyzed. AC/DC shimming provide the highest correlation (R^2^ = 0.98, P = 0.001) with ground-truth values for metabolite concentration. Clinical translation of AC/DC and MRSI is demonstrated in a patient with mutant-IDH1 glioma where it enables imaging of D-2-hydroxyglutarate oncometabolite with a 2.8-fold increase in contrast-to-noise ratio at higher resolution and more brain coverage compared to previous 7 T studies. Hence, AC/DC technology may help ultra-high field MRSI become more feasible to take advantage of higher signal/contrast-to-noise in clinical applications.

## Introduction

Magnetic resonance spectroscopic imaging (MRSI) at ultra-high field (≥ 7 T) has the potential to map the neurochemistry of the human brain with high spatial resolution and fast acquisition times^1^. This may be possible due to the increase in sensitivity^2^ with the strength of the static magnetic field B_0_. In addition, the ultra-high B_0_ field provides more specificity for an extended metabolic profile due to increased spectral dispersion^2^. However, in practice the realization of this performance is challenging because distortions of the B_0_ field induced by susceptibility anisotropy at air-tissue interfaces^3^ also scale with the field strength. With the recent clinical adoption of 7 T MR scanners it is imperative to provide technical solutions that enable robust performance and translation of MRSI for metabolic imaging in patients.

A homogeneous B_0_ field is critical for high quality MRSI data^4^, and determines the spectral linewidth (LW) and signal to noise ratio (SNR). Adequate LW and SNR are required for reliable spectral fitting for precise and accurate metabolite quantification. Homogeneous B_0_ field, narrow LW (< 0.1 ppm) and high SNR (> 10) are difficult to obtain uniformly across the brain, especially at ultra-high field. Brain areas that are close to air cavities such as the anterior part of the frontal lobe or close to the skull base are particularly affected by rapidly changing and spatially complicated B_0_ field distributions. Furthermore, improving B_0_ homogeneity during MRSI acquisition can improve water suppression^5,6^ or spectral editing^7,8^ that rely on frequency selective excitation, hence reducing imaging artifacts associated with imperfect water suppression or spectral editing.

Mitigation of B_0_ inhomogeneity is achieved by B_0_ shimming methods^9^ that create an opposite B_0_ field pattern to that induced by the human head, thus cancelling the B_0_ distortions across the brain. A key factor in the performance of B_0_ shimming is the hardware that creates the opposite B_0_ field pattern, along with software algorithms to measure and calculate this pattern. Two types of B_0_ shim hardware exist: (1) traditionally, large shim coils that are modeled on a spatial spherical harmonics (SH) distribution^10–13^, and (2) recently, small shim coils placed in an array configuration^14–16^ that provide a more arbitrary spatial distribution. This hardware can be run either in static or dynamic shimming^17^ mode. Currently, human MR scanners are equiped with spherical harmonics shimming, which in clinical configuration include up to eight shim channels for first and second order spherical harmonics, with some 7 T research systems equipped with a few 3rd-order SH shim coils.

Custom shim inserts with up to 5th order spherical harmonics for static B_0_ shimming were shown^12^ to significantly improve MRSI of human brain at 7 T. Slice-specific dynamic shim update for up to 3rd-order spherical harmonics was shown^18^ to improve MRSI at 7 T. Dynamic shimming with multi-coil arrays^15,16,19^ have been also shown to improve B_0_ shimming at ultra-high field through additional degrees of freedom. In addition to shimming hardware, software algorithms for B_0_ fieldmapping^20,21^ , B_0_ shimming^20,22,23^ and B_0_ correction^23,24^ have been shown to improve the homogeneity of the B_0_ field by better phase unwrapping for fieldmap calculation, excluding scalp areas or outliers in the fieldmaps, and advanced MRSI reconstruction.

More recently^25–27^, a hybrid hardware with integrated RF-receive and B_0_-shim arrays using the same loop coils that simultaneously run AC and DC currents (AC/DC coils) was introduced to take advantage of the array design for both sensing (B_1-_) and shimming (B_0_) magnetic fields. The hybrid AC/DC coils offer several advantages: (1) compact design with minimal additional hardware that is easy to integrate with the MR scanners, (2) low inductance DC coils which can be switched rapidly using low-cost, low-voltage amplifiers^28^, and (3) the DC coils are away from conductive structures of the magnet and can be switched without inducing eddy currents. In addition, the number of AC/DC channels can be easily increased with minimal increase in coil dimension and weight, while for spherical harmonics adding high-order channels significantly increases dimension and weight of the shim insert. When combined with custom shim optimization software, integrated AC/DC coils provide additional degrees of freedom to shape the B_0_ field especially in brain regions close to air-tissue interfaces that are the most affected by susceptibility anisotropy.

Here, we investigate for the first time the use of a novel AC/DC 31-channel coil to improve the performance of MRSI in human brain at 7 T ultra-high field. Many MRSI studies in the brain at 7 T^29–32^ have been performed using single slice or thin slabs covering the centrum semiovale due to insufficient B0 shimming for larger brain volumes. 2SH shimming cannot homogenize local magnetic fields within each MRSI voxel over the whole-brain within an acceptable linewidth range, thus limiting the number of usable MRSI voxels. We hypothesize that the AC/DC shimming superimposed on the scanner’s standard second order shimming (2SH) will provide adequate spectral quality to perform 3D MRSI over a thick whole-brain slab at 7 T. The advantage of AC/DC shimming for 3D MRSI was demonstrated at 3 T^33,34^ , and a greater benefit is expected at higher fields.

A key clinical application of MRSI has emerged in neuro-oncology and neuro-surgery by imaging the oncometabolite D-2-hydroxyglutarate (2HG)^35^ in mutant isocitrate dehydrogenase (IDH-1,2) gliomas^36^, that hence may be used for glioma sub-typing^37^. To date 2HG has been shown to be a highly sensitive and specific imaging biomarker to diagnose^38,39^, prognosticate^40^, plan treatment^41^ and predict response to targeted therapy^42^ in mutant IDH glioma patients. Although, production of 2HG occurs only within mutant IDH tumor and it is virtually undectable outside the tumor, measuring 2HG by MRSI is challenging because the chemical shits of its five protons^43^ have spectral overlap with abundant brain metabolites such as glutamate and glutamine^44^. At 7 T ultra-high field there is increased SNR and peak separation of metabolite spectra compared to 3 T clinical field, however these advantages can be fully exploited only if sufficiently narrow linewidth can be obtained. Optimized pulse sequences for 2HG detection have been developed for 7 T, however to date they have been demonstrated either for single voxel^45,46^ or single slice spectroscopy^47^ with rectangular volume selection. The increased spectral dispersion and SNR by ultra-high field has been exploited even at 9.4 T^48^ using STEAM volume excitation with 2D phase-encoded MRSI to detect 2HG in glioma patients. Though it is easier to obtain a homogeneous B_0_ field over a small volume, such limited excitation schemes may only partially cover the tumor and the healthy brain, hence missing the full potential of imaging to probe tumor margings and tumor heterogeneity. With the improvement in B_0_ homogeneity provided by AC/DC shimming such limitations could be removed at ultra-high field.

Hence, our first aim for AC/DC shimming was to obtain an MRSI protocol that provides unrestricted brain coverage in the axial view combined with 3D slice (phase) encoding. A second aim was to increase spatial resolution and speed-up acquisition, for which we used spectral-spatial encoding with a stack of spiral out-in^49,50^ k-space trajectories. Most fast spectroscopic and imaging sequences based on spiral and echo-planar trajectories may suffer from off‐resonance effects due to inhomogeneous B_0_ at ultra-high field^9,51^. Improving B_0_ homogeneity with AC/DC shimming could thus reduce spiral imaging artifacts at 7 T. Third, to improve the robustness of our MRSI method we employed an interleaved volumetric EPI navigator^52–54^, which can monitor in real-time the stability of AC/DC and 2SH shimmings and perform frequency and motion correction. By combining all of the above methods, we sought to obtain a 3D MRSI protocol at 7 T that provides more brain coverage, higher spatial resolution, faster acqusition times and reliable quantification for 2HG and other brain metabolites compared to previous work^47^.

## Results

### Simulations

**Figure 1:**
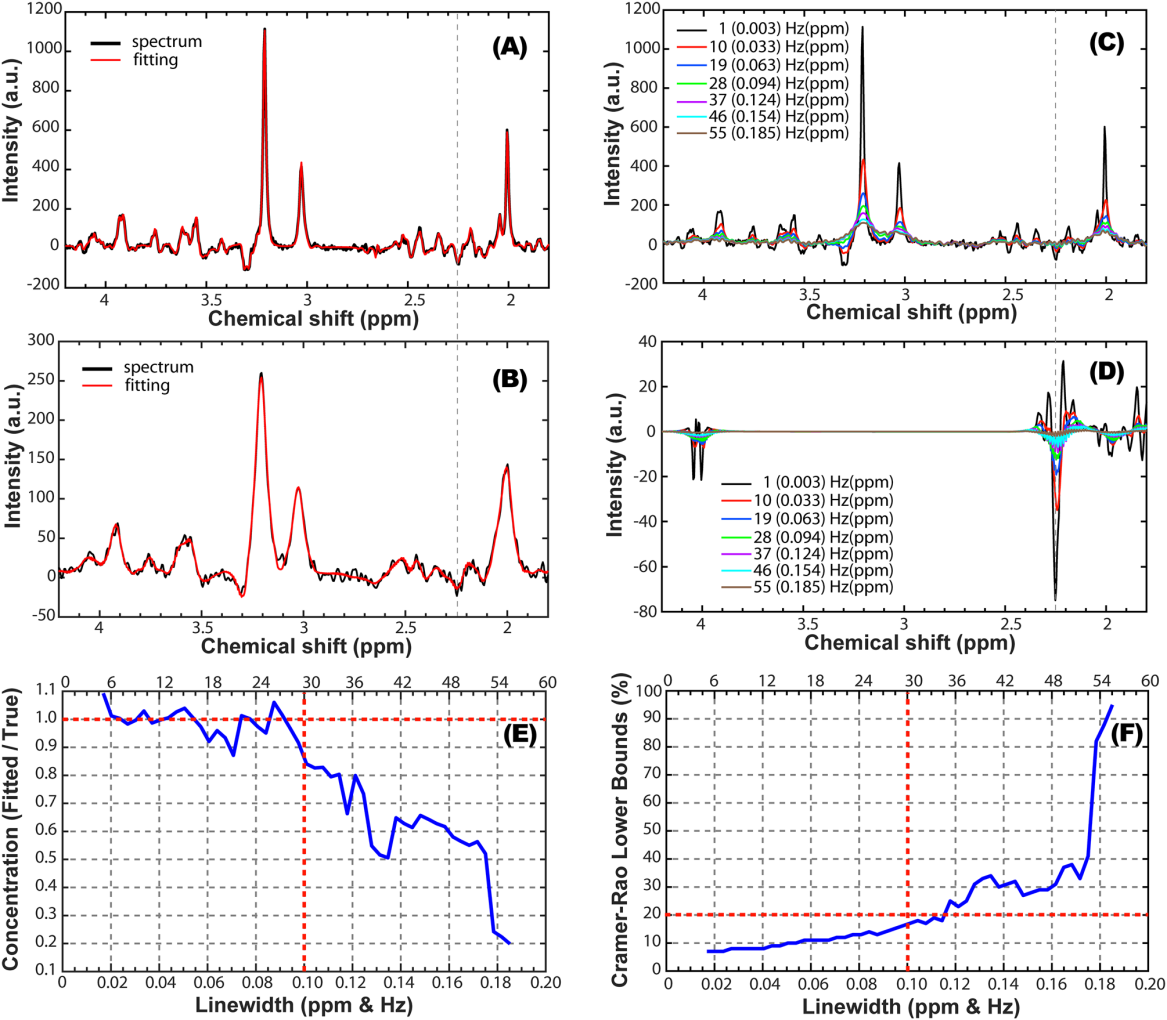
Simulations of brain spectra for 7 T and ASE excitation (TE1/TE2 = 50/28 ms) under increasing linewidths. **(A)** Simulated (black) and fitted (red) spectrum for LW = 3 Hz (0.01 ppm); **(B)** Simulated (black) and fitted (red) spectrum for LW = 30 Hz (0.1 ppm); **(C)** Overlay of simulated brain spectra for increasing linewidth; **(D)** Overlay of 2HG spectra fitted by LCModel in the simulated brain spectra for increasing linewidth; **(E)** Accuracy of 2HG fitting for increasing linewidth (1 ± 0.1 fit/true is considered acceptable interval; true value as dashed horizontal red line, vertical red line the maximum acceptable linewidth); **(F)** Precision of 2HG fitting for increasing linewidth (CRLB < 20% is considered acceptable precision; 20% marked by dashed horizontal red line, vertical red line the maximum acceptable linewidth). The vertical dashed line at 2.25 ppm in (**A**–**D**) indicates the position of the negative 2HG peak for TE = 78 ms.

The effects of increasing linewidths on the spectral fitting and 2HG quantification are shown in Fig. 1 for simulated spectra. For narrow linewidths ≤ 0.1 ppm, a rich spectral pattern can be noticed in the 2.1–2.6 ppm range (Fig. 1A,C). For linewidths above 0.1 ppm this spectral pattern is gradually lost by partial signal cancelation due to increasing overlap of negative and positive peaks (Fig. 1B). The characteristic negative peak of 2HG obtained for double spin-echo TE1/TE2 = 58/20 ms is clearly visible at 2.25 ppm for linewidths up to 0.1 ppm (Fig. 1A,C,D). The ratio between estimated and true 2HG concentration (Fig. 1E) is close to 1 (± 0.1 range) for linewidths ≤ 0.1 ppm, and rapidly decreases towards 0 for increasing linewidths. The relative CRLB (Fig. 1F) remains under 20% for linewidths ≤ 0.1 ppm, but sharply increases for larger linewidths.

### Phantoms

**Figure 2:**
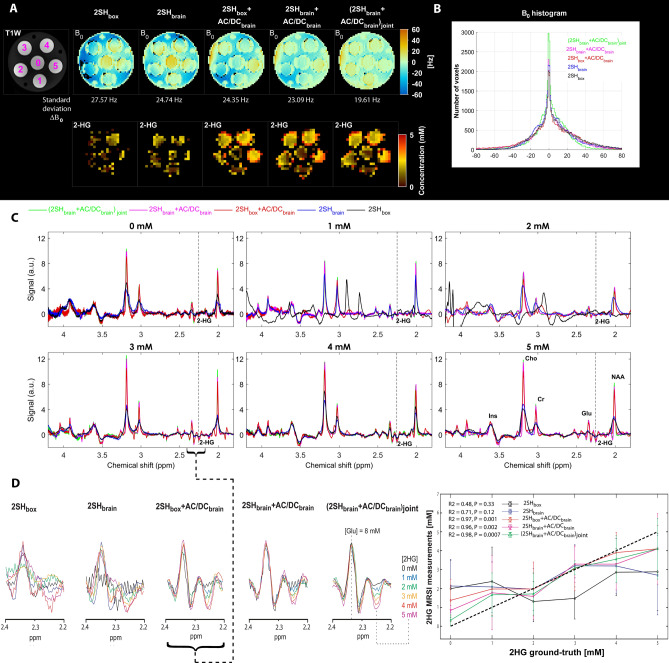
Phantom measurements performed under five shimming conditions 2SH_box_, 2SH_brain_, 2SH_box_ + AC/DC_brain_, 2SH_brain_ + AC/DC_brain_, and (2SH_brain_ + AC/DC_brain_)_joint_. **(A)** B_0_ fieldmaps maps of 2-HG and T1-weighted MRI with concentration of 2-HG marked on every tube 0, 1, 2, 3, 4, 5 mM (magenta). **(B)** Histogram distribution of the B_0_ fieldmaps for all shimming conditions. **(C)** spectra from each tube for each shimming method overlaid, the negative 2-HG peak at 2.25 ppm is indicated by vertical dashed lines. **(D)** Zoom on the 2.2–2.4 ppm spectral range that contains the positive glutamate peak at 2.35 ppm and negative 2HG peak at 2.25 ppm. Correlation analysis between fitted and ground-truth 2HG concentration for all shim conditions.

The results of the five different shimming conditions in the phantom are shown in Fig. 2. The B_0_ fieldmaps (Fig. 2A) at the top show gradual homogeneity improvement going from 2SH_box_ to (2SH_brain_ + AC/DC_brain_)_joint_ shimming, with 29% narrower width of B_0_ histograms (Fig. 2B) that decreased from 27.57 Hz (0.093 ppm) for 2SH_box_ to 19.61 Hz (0.066 ppm) for (2SH_brain_ + AC/DC_brain_)_joint_ shimming. The 2HG maps (Fig. 2A) correspond better to the T1 weighted image and the known 2HG concentration in the tubes, when AC/DC shimming is superimposed on the 2SH shimming. Examples of spectra (Fig. 2C) from each tube show narrower lines and less artifacts for measurements with superimposed AC/DC shimming. In particular, artifacts coming from insufficient water suppresion and frequency shifts are visible in spectra acquired with 2SH_box_ shimming in the 1 mM and 2 mM tubes that have the worst local B_0_ distribution. In Fig. 2D is shown zoom on the spectral interval [2.2, 2.4] ppm containing the 2HG negative peak at 2.25 ppm and the Glu positive peak at 2.35 ppm. The 2SH + AC/DC shims show a clear titration with concentration for the negative 2HG peak at 2.25 ppm and a stable positive Glu peak at 2.35 ppm for constant Glu concentration, while there is more variability in the case of the 2SH shims. Correlation between the measured and ground-truth 2HG concentrations is shown in the right most panel of Fig. 2D. The highest correlation coefficient and statistical significance (R2 > 0.95, P < 0.05) is obtained for the three combinations of 2SH + AC/DC shims, while the 2SH only shims provide only moderate correlation without statistical significance.

### Human subjects

**Figure 3:**
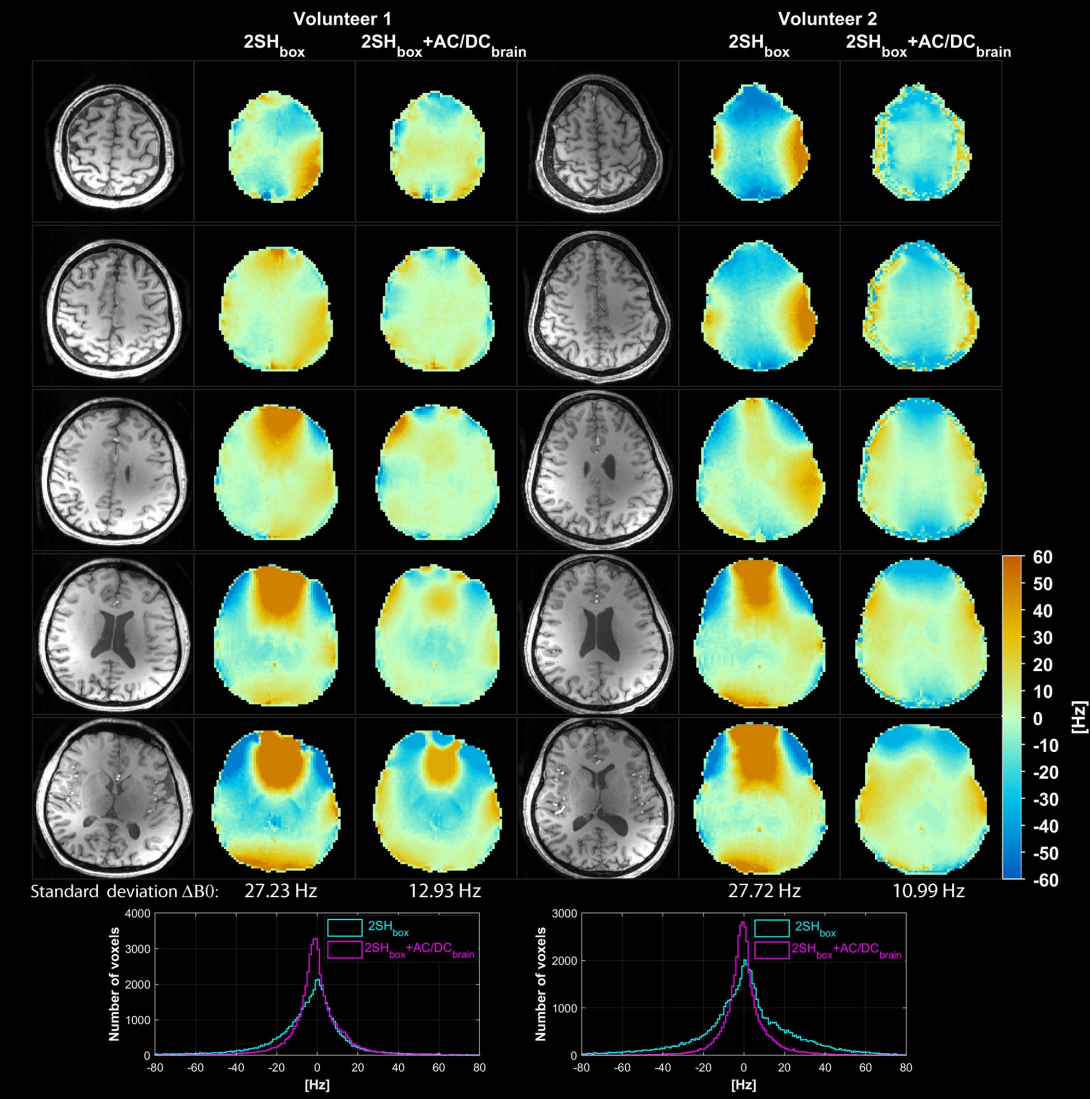
Comparison of B_0_ homogeneity obtained with 2SH_box_ and 2SH_box_ + AC/DC_brain_ shimmings in the first two healthy volunteers. Five representative slices are shown throughout the shimmed brain slab, with the standard deviation of B_0_ distribution indicated below. Histograms of B_0_ distribution over the entire brain slab are shown overlaid at the bottom for both shimming methods. MEMPRAGE anatomical images are shown in the left most columns for each volunteer.

**Figure 4:**
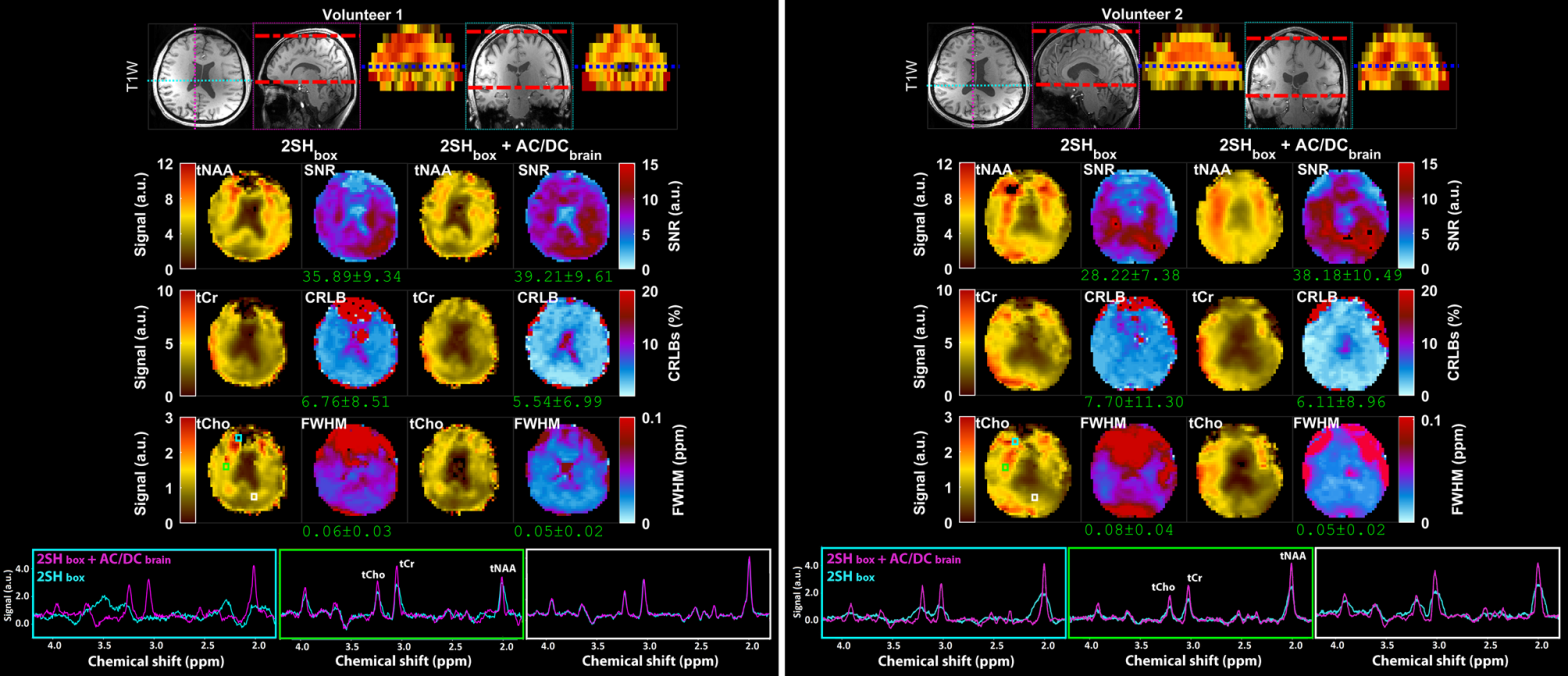
Comparison of MRSI data obtained with 2SH_box_ and 2SH_box_ + AC/DC_brain_ shimmings in the first two healthy volunteers. An inferior axial slice is shown from the stack of 3D MRSI data as indicated by the blue dashed line in the coronal and sagital views at the top. The metabolic maps of total NAA (tNAA), total Choline (tCho), total Creatine (tCr), linewidth (FWHM), signal-to-noise ratio (SNR), and Cramer-Rao lower bounds (CRLB of tCr) are shown for both shimming methods. Examples of spectra from frontal (cyan box), central (green box), and occipital (white box) regions are shown overlaid for 2SH_box_ (cyan) and 2SH_box_ + AC/DC_brain_ (magenta). The values under the maps indicate the mean and standard deviation calculated over the whole-brain slab. MEMPRAGE anatomical images are shown at the top.

**Figure 5:**
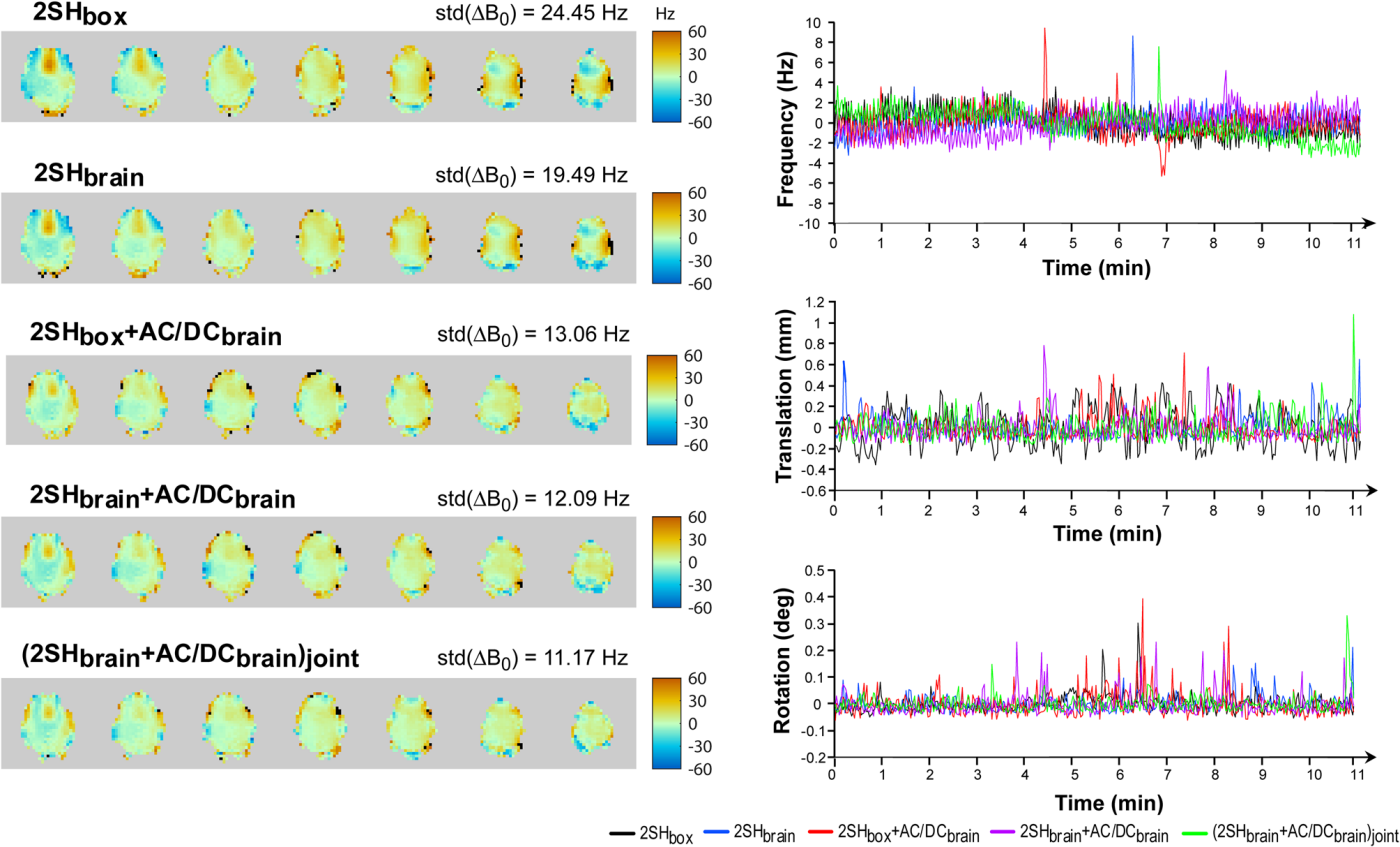
Real-time motion correction and frequency correction for 3D MRSI in the third healthy volunteer under five shimming conditions 2SH_box_, 2SH_brain_, 2SH_box_ + AC/DC_brain_, 2SH_brain_ + AC/DC_brain_, and (2SH_brain_ + AC/DC_brain_)_joint_. Left panel: real-time B_0_ fieldmapping for all shimming conditions with the standard deviation of B_0_ distribution indicated on the right side of the map title. Right panel: real-time plots of the frequency (up), translation (middle), and rotations (bottom).

**Figure 6:**
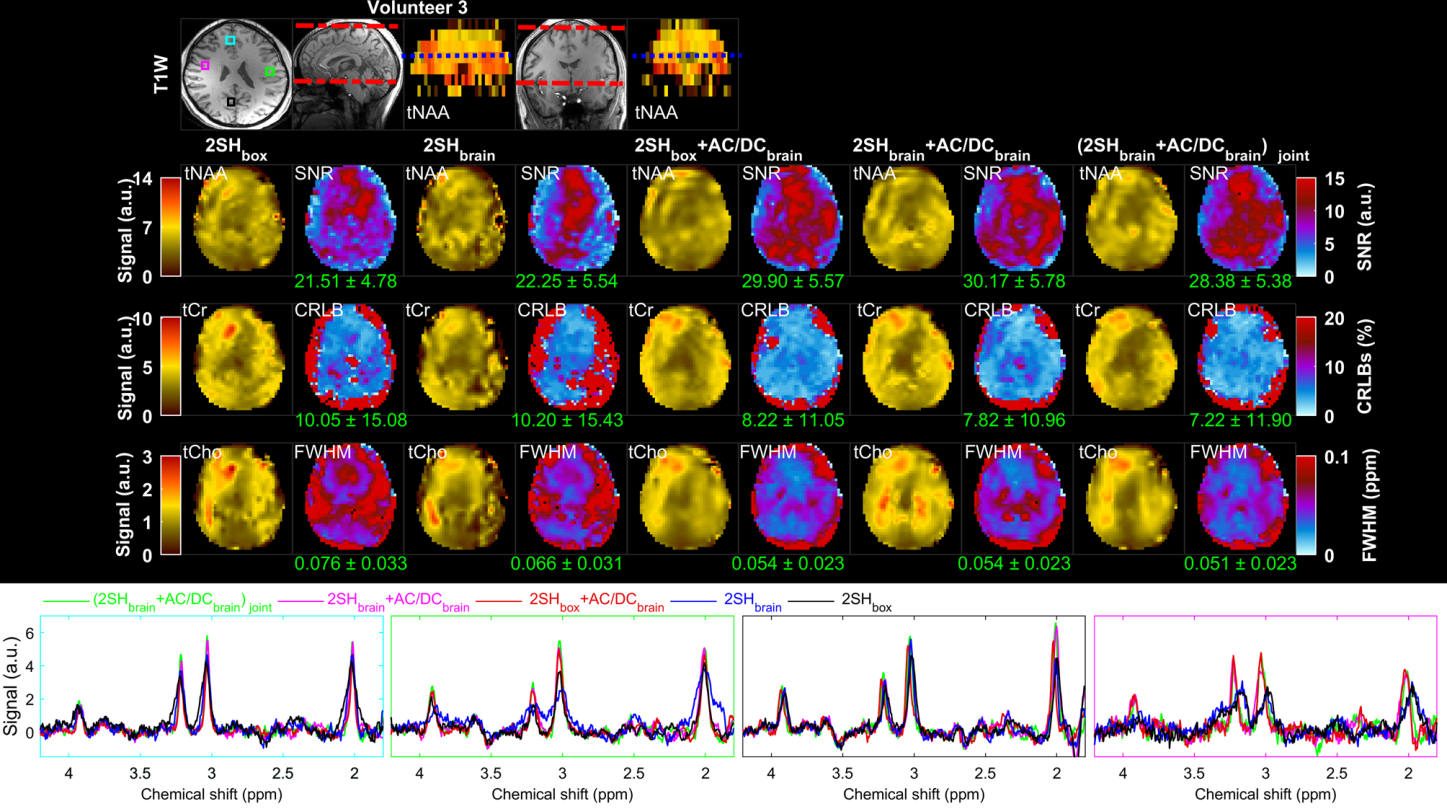
Comparison of MRSI in the third healthy volunteer under five shimming conditions 2SH_box_, 2SH_brain_, 2SH_box_ + AC/DC_brain_, 2SH_brain_ + AC/DC_brain_, and (2SH_brain_ + AC/DC_brain_)_joint_. An inferior axial slice is shown from the stack of 3D MRSI data as indicated by the blue dashed line in the coronal and sagital views at the top. The metabolic maps of total NAA (tNAA), total Choline (tCho), total Creatine (tCr), linewidth (FWHM), signal-to-noise ratio (SNR), and Cramer-Rao lower bounds (CRLB of tCr) are shown for all shimming methods. Examples of spectra from frontal (cyan box), left lateral (green box), right lateral (pink box), and occipital (black box) voxels are shown overlaid for all shimming conditions. The values under the maps indicate the mean and standard deviation calculated over the whole-brain slab. MEMPRAGE anatomical image is shown at the top, with the limits of the MRSI slab shown on the sagital and coronal slices by the red dashed lines.

**Figure 7:**
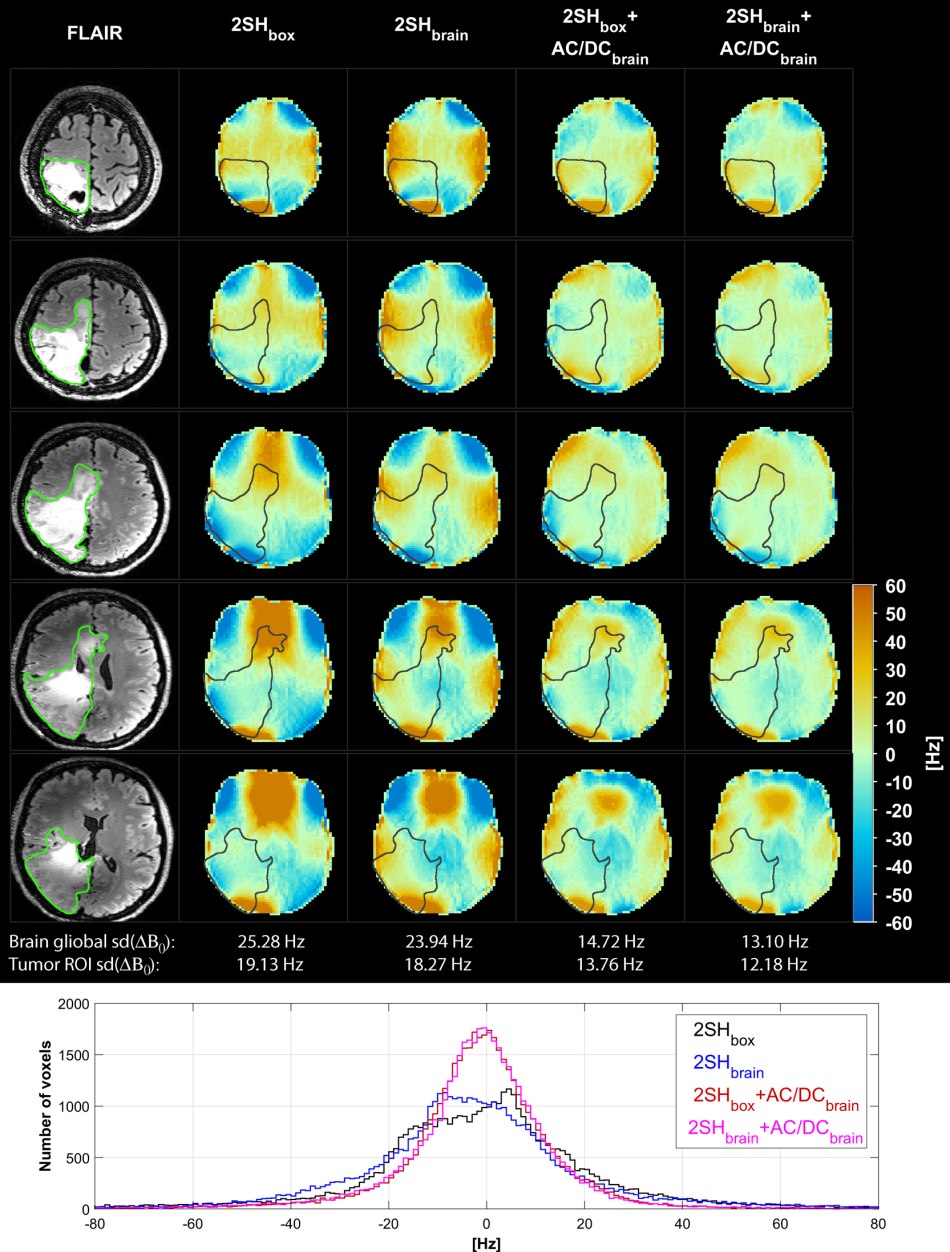
Comparison of B_0_ fieldmaps in mutant IDH1 glioma patient under four shimming conditions 2SH_box_, 2SH_brain_, 2SH_box_ + AC/DC_brain_, and 2SH_brain_ + AC/DC_brain_. Five representative slices are shown through the shimmed whole-brain slab with the tumor ROI contours. Standard deviation of B_0_ distributions is given below for the entire slab and the tumor. Histograms of B_0_ distribution over the entire brain slab are shown overlaid at the bottom for all the shimming conditions. FLAIR anatomical image is shown on the left most column.

**Figure 8:**
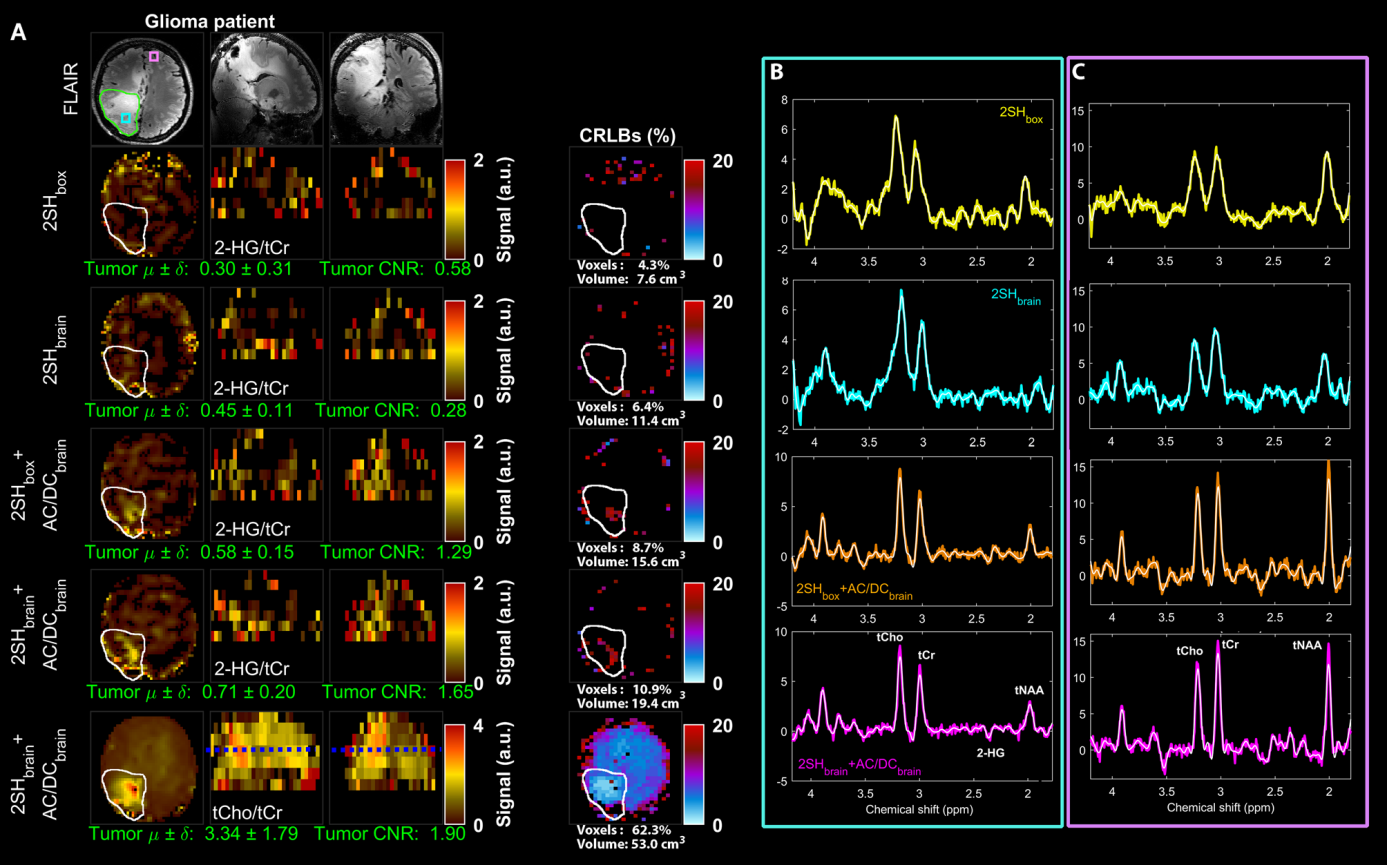
Maps of the D-2-hydroxyglutarate to total creatine (2-HG/tCr) shown in axial, sagital, coronal views in the mutant IDH1 glioma patient obtained with the four shimming conditions 2SH_box_, 2SH_brain_, 2SH_box_ + AC/DC_brain_, and 2SH_brain_ + AC/DC_brain_. FLAIR image is shown at the top, and the map of total choline to total creatine (tCho/tCr) for 2SH_brain_ + AC/DC_brain_ shim is shown at the bottom. Cramer-Rao lower bounds (CRLB) maps are shown at the right of the metabolic maps. Below each metabolic map the mean and standard deviation of metabolic ratios in the tumor are calculated, and the contrast-to-noise ratio of the tumor. The tumor contours are indicated on the FLAIR and metabolic maps. The tumor 2HG volume detected with CRLB < 20% and the ratio of 2HG to FLAIR volume is indicated for each shimming under the CRLB maps. Examples of spectra from the tumor (B) and healthy brain (C) are shown for all shimming conditions in the right two columns. The maps of tNAA, tCho, tCr, SNR, FWHM and CRLB (of tCr) are shown in Supplementary Fig. 4.

In vivo results are presented in Figs. 3, 4, 5, 6, 7, 8 and summarized in Table 1. B_0_ fieldmaps obtained with the GRE sequence in two healthy volunteers with 2SH_box_ and 2SH_box_ + AC/DC_brain_ shims are shown in Fig. 3. Improvement in B_0_ homogeneity is visible for 2SH_box_ + AC/DC_brain_ shimming, with marked regional improvements in the anterior frontal lobe. The histograms of B_0_ values show 57% narrower distribution for 2SH_box_ + AC/DC_brain_ shimming (12.93 Hz and 10.99 Hz) compared to 2SH_box_ shimming (27.23 Hz and 27.72 Hz).

Results from MRSI measurements for the same two volunteers and the same two shimming conditions are shown in Fig. 4. The metabolic maps of total NAA, choline and creatine show better agreement with the anatomical imaging and less artifacts for 2SH_box_ + AC/DC_brain_ shimming, which are particularly visible in 2SH_box_ as missing areas due to signal dropout in the anterior frontal regions. The quality parametric maps (SNR, CRLB, FWHM) show an increase in mean SNR by 9–35%, a decrease of mean CRLB by 18–21%, and a decrease of mean linewidth by 17–37% for 2SH_box_ + AC/DC_brain_ compared to 2SH_box_. Local improvements in quality parametric maps are even higher for the anterior frontal brain areas. Overlay of spectra from voxels in anterior, middle and posterior brain areas show more narrower metabolic peaks with reduced lineshape distortion for 2SH_box_ + AC/DC_brain_ shimming. In volunteer one the 2SH_box_ spectra in the anterior frontal regions have large frequency shifts that cannot be corrected by the LCModel frequency correction, while this is not a problem for 2SH_box_ + AC/DC_brain_ spectra. Additional axial slices from the 3D MRSI brain slab are shown in Supplementary Figs. S1 and S2.

In Fig. 5 the real-time B_0_ fieldmaps obtained with the navigator are shown for the third healthy volunteer and all five shimming conditions. The B_0_ fieldmaps show a gradual improvement in homogeneity, up to 55% from a standard deviation of 24.45 Hz (0.082 ppm) for 2SH_box_ shimming to a standard deviation of 11.17 Hz (0.037 ppm) for (2SH_brain_ + AC/DC_brain_)_joint_ shimming. The plots of frequency correction show similar stability for the scanner shimming hardware (2SH) alone and the combined scanner plus AC/DC shimming hardware (2SH + AC/DC). Isolated spikes in the frequency plots correspond to brief head motion as shown in the translation and rotation plots. The B_0_ fieldmaps obtained with the GRE sequence are presented in Supplementary Fig. 3, which also shows that B_0_ histograms have narrower width for shim conditions where AC/DC is superimposed on 2SH.

The metabolic maps and the quality parametric maps obtained in volunteer 3 for all five shimming conditions are shown in Fig. 6. All the metabolic maps obtained with the three combinations of 2SH + AC/DC shims show better agreement with anatomical imaging and less artifacts compared to the two 2SH only shims. The quality parametric maps show mean improvements of 25% increased SNR, 19% decreased CRLB, and 18% narrower FWHM, between 2SH only and 2SH + AC/DC shimmings.

Figure 7 shows the B_0_ fieldmaps obtained with the GRE sequence in the mutant IDH1 glioma patient under four shimming conditions. Compared to the 2SH_box_ the global B_0_ homogeneity improves by 5.5% for 2SH_brain_, by 42% for 2SH_box_ + AC/DC_brain_, and by 48% for 2SH_brain_ + AC/DC_brain_ shims, respectively. Marked improvements in local B_0_ homogeneity are also obtained for the tumor region of interest (ROI) by 4.5%, 28% and 36%, respectively, when comparing the same shim conditions.

The tumor specific maps of 2HG to total creatine (2HG/tCr)^41^ obtained in the mutant IDH1 glioma patient are shown in Fig. 8. The tumor is not diagnostically apparent in the 2HG/tCr maps obtained with the two 2SH shimmings (2SH_box_ and 2SH_brain_), which show no clear increase in this metabolic marker above background. The 2HG/tCr maps obtained with two combinations of 2SH + AC/DC shims (2SH_box_ + AC/DC_brain_ and 2SH_brain_ + AC/DC_brain_) show an increase in this metabolic marker within the tumor ROI. The CRLB maps for 2HG fitting show the largest clusters of tumor voxels having CRLB < 20% and fewer false positive voxels outside the tumor for measurements obtained with 2SH_box_ + AC/DC_brain_ and 2SH_brain_ + AC/DC_brain_ shims. The improved 2HG quantification provides fewer false negative voxels in the tumor and fewer false positive voxels outside the tumor, resulting in better image quality with higher tumor contrast-to-noise ratio for 2SH_box_ + AC/DC_brain_ and 2SH_brain_ + AC/DC_brain_ shimmings. By comparison, the maps obtained with 2SH shims are non-diagnostic with a contrast-to-noise ratio less than one, indicating tumor 2HG signal below the background variability. Tumor heterogeneity is visible in the metabolic maps of 2HG/tCr and tCho/tCr (total-choline/creatine) obtained by 2SH_brain_ + AC/DC_brain_. Examples of spectra from tumor and healthy brain show improvement in spectral resolution with better peak separation, higher SNR, and less baseline artifacts for 2SH_box_ + AC/DC_brain_ and 2SH_brain_ + AC/DC_brain_ shims.

Metabolic maps of tNAA, tCho, tCr and the quality parametric maps SNR, FWHM and CRLB from mutant IDH1 glioma patient are presented in Supplementary Fig. 4. The metabolic maps obtained with the two 2SH + AC/DC shimmings show the tumor more clearly, with less image artifacts compared to the two 2SH shims. In particular, total choline as a tumor marker shows a marked increase in the tumor only for 2SH + AC/DC maps, while areas of high choline far from tumor are visible in 2SH maps. When compared to 2SH_box_ the: (1) SNR increases by 3% for 2SH_brain_, by 14% for 2SH_box_ + AC/DC_brain_, and by 17% for 2SH_brain_ + AC/DC_brain_; (2) CRLB decreases by 4% for 2SH_brain_, by 17% for 2SH_box_ + AC/DC_brain_, and by 23% for 2SH_brain_ + AC/DC_brain_; (3) FWHM decreases by 10% for 2SH_brain_, by 13% for 2SH_box_ + AC/DC_brain_, and by 18% for 2SH_brain_ + AC/DC_brain_. Quantitative results from in vivo measurements in healthy subjects and patients are summarized in Table 1.

**Table 1:** Mean and standard deviation of B_0_-maps, MRSI spectral linewidth (FWHM), signal-to-noise ratio (SNR), and goodness of fit measure by Cramer-Rao lower bounds (CRLB of tCr) metric, contrast-to-noise ratio (CNR of 2HG), the percentage of voxels with acceptable quality (i.e. FWHM < 0.1 ppm, CRLB < 20%, SNR > 10).

	ΔB_0_ (Hz)	FWHM (ppm)	SNR	CRLB (%)	CNR	Voxels (%)
*2SH_box_	26.17 ± 1.56	0.071 ± 0.032	21.64 ± 6.95	10.47 ± 15.43	0.58	78.38 ± 7.39
^#^2SH_brain_	21.40 ± 1.29	0.069 ± 0.033	22.35 ± 6.08	10.12 ± 15.34	0.28	84.45 ± 4.60
*2SH_box_ + AC/DC_brain_	14.24 ± 3.03	0.053 ± 0.021	27.83 ± 7.72	8.85 ± 12.03	1.29	93.43 ± 5.36
^#^2SH_brain_ + AC/DC_brain_	13.34 ± 2.00	0.059 ± 0.024	33.26 ± 5.65	8.54 ± 11.52	1.65	93.55 ± 0.64
^§^(2SH_brain_ + AC/DC_brain_)_joint_	11.17	0.051 ± 0.023	28.38 ± 5.38	8.22 ± 11.99	NA	95

The CNR was calculated for tumor 2HG in the mutant IDH1 patient (*NA* not acquired).

*This shim was measured in four subjects.

^#^This shim was measured in two subjects.

^§^This shim was measured in one subject.

## Discussion

In this work we demonstrated that an integrated AC/DC shim array improves 3D MRSI at 7 T over a whole-brain slab when multi-coil array shimming is combined with the standard scanner second order spherical harmonics shimming. The improvements are particularly large in the anterior frontal areas and inferior slices, which are hard to shim with the standard 2SH method, but improvements are obtained thoroughout the brain. We investigated also the improvements due to optimization of 2SH shimming over the head vs brain, with the former being the manufacturer’s method which includes the brain, skull and scalp in the shimmed volume. The 2SH shimming over the brain slab (2SH_brain_) improves B_0_ and MRSI compared to the 2SH shimming over the head slab (2SH_box_), however these improvements are smaller than what is obtained by adding the AC/DC shimming to the 2SH shimming for either shim optimization (box or brain). Hence, the improvements by AC/DC shimming are largely due to the ability of the AC/DC coil to generate highly arbitrary B_0_ field patterns, and secondarily due to optimizing shimming over the brain only compartment. All three 2SH + AC/DC shims that were investigated provided comparable improvements, with a slightly superior performance for the jointly optimized (2SH_brain_ + AC/DC_brain_)_joint_. The results from humans are summarized in Table 1.

The AC/DC shimming improved both global and local B_0_ homogeneity as measured by narrower histogram distributions (55%) and spectral linewidths (29%), respectively. Global B_0_ homogeneity determines how much the mean frequency of a certain MRSI voxel is shifted from the central frequency in the shim volume, and is important for frequency selective schemes such as water suppression^5,6^ and spectral editing^7^. Adjustment of water suppression and spectral editing is performed globally on the central frequency in the brain, and for MRSI voxels that are shifted far from the central frequency the suppression and editing efficiency is compromised. This can lead to large residual water signal that overwhelms the metabolite signal in those voxels, but can also contaminate other voxels due to reduced point spread function of MRSI. This is true not only for water suppression schemes such as WET^5^, but also for metabolite cycling^6^ that is dependent on frequency selective metabolite inversion for water subtraction. Insufficient water suppression manifests as large baseline artifacts that distort spectra and interfere with metabolite fitting. Such artifacts were visible in phantom and in-vivo MRSI measurements with 2SH shimmings, and were largely reduced by the addition of AC/DC shimming. In phantom the susceptibility difference between magnevist-doped (paramagnetic) solutions inside the tubes and the magnevist-free (diamagnetic) surrounding solution cannot be corrected well by 2SH shimming, but this is reduced by AC/DC shimming. Further improvement in coil combination^55,56^ and B_1_ transmit efficiency^57^ can improve spatial correlation of metabolic maps at ultra-high field. Local B_0_ homogeneity determines the intravoxel spectral linewidth in MRSI, which is important for spectral peak separation and metabolite fitting. AC/DC shimming provided a significant reduction (29%) in spectral linewidth and an increase (31%) in SNR, which resulted in improved accuracy for metabolite quantification by smaller (22%) Cramer-Rao lower bounds. While local homogeneity might be improved by smaller voxels with high resolution MRSI^58^, such acquisitions may not always be possible due to SNR constraints for low concentration metabolites (GABA, GSH), and better shimming may also improve high resolution data. On the other hand, challenges for water suppression and spectral editing due to global inhomogeneity do not change with image resolution and cannot be corrected with post-processing B_0_ correction methods^24^, hence requiring better shimming. Furthermore, better global and local B_0_ homogeneity can help MRSI in the case of spectral-spatial encoding^59^ and advanced low-rank reconstruction methods^60^.

Multi-coil shimming has been explored for single voxel spectroscopy at ultra-high field at 9.4 T^16^, however to date the use of shim arrays for MRSI has not been explored beyond 3 T^33,34^. The MRSI improvements obtained by us at 7 T with AC/DC shimming are in line with previously reported improvements using 4^th^-order spherical harmonics shimming^12^. Hence, AC/DC shimming may provide an alternative to high-order spherical harmonics shimming, with lighter in-bore hardware. The stability of our AC/DC hardware was monitored by real-time B_0_ fieldmapping with a volumetric navigator which showed similar frequency drift for 2SH and 2SH + AC/DC shimmings. With further integration of AC/DC and navigator the real-time fieldmapping could be used for shim update in case of subject motion as shown for the 2SH shimming^52,53^.

The AC/DC methodology has potential for clinical translation as demonstrated in the mutant IDH1 glioma patient. The AC/DC shimming enabled us to obtain the largest brain coverage shown to date for 2HG imaging at 7 T. The quality of 2HG imaging was improved by AC/DC shimming with fewer voxels with false results, which yielded higher tumor contrast-to-noise ratio. This patient was particularly challenging because of prior tumor surgery and chemotherapy that may have decreased the 2HG levels, but also due to metal implants used for skull repair. The metal implants can interfere with B_0_ shimming and B_1_ transmit efficiency which can decrease the SNR of MRSI. It was hence critical to improve the SNR for 2HG detection by using the AC/DC shimming. The in-vivo 2HG results are in line with results from the calibration phantom that showed the highest correlation between the measured and ground-truth 2HG concentrations, and from simulations which predicted that precision and accuracy of 2HG quantification is dependent on the linewidth. Our 3D MRSI sequence has three advantages over previous 2D MRSI^47^ used for 2HG detection at 7 T: (1) compared to PRESS used in Ref.^47^, our ASE excitation has 14-times less chemical shift displacement error for slab localization with a sharp profile, and also compensates for B_1_ transmit inhomogeneity; (2) our efficient spectral-spatial encoding using spiral out-in allowed us to obtain the highest spatial resolution so far reported for 2HG imaging at 7 T and in a shorter acquisition time; (3) our whole-brain slab coverage and high resolution metabolic imaging makes possible to probe the full spatial extent and heterogeneity of tumor metabolism, which complements better anatomical imaging. Compared to semi-adiabatic single voxel excitation shown at 7 T for 2HG detection^46^, our ASE uses fewer pulses reducing specific absorption rate and echo time to allow faster repetition times and increased SNR.

Our study has some limitations. We limited the AC/DC shimming to a brain slab of 70 mm thickness because there is less gain in B_0_ homogeneity for thicker slabs. Our AC/DC coil used a design that was optimized for detection of RF signal, rather than to generate local B_0_ field patterns, but improved versions may be designed in the future that are optimized for thicker brain slabs. Our study was also limited by a small sample size of four subjects and further validation of this methodology may be needed in larger studies. However, we obtained reproducible and consistent improvements in all subjects and the calibration phantom.

In summary, AC/DC shimming significantly improves B_0_ homogeneity at 7 T, enabling MRSI to take full advantage of higher SNR available at ultra-high field. Hence, AC/DC shimming provides better 3D MRSI data quality and metabolite quantification for imaging human brain metabolism at 7 T. This methodology may increase the number of successful imaging investigations and reduce false results. Hence, AC/DC can increase the throughput of 7 T without the need for repeated scans after failed examinations, which may save costs in clinical operation and increase patient comfort. Robustness is further enhanced by the use of a navigator for real-time motion correction and frequency update. This robust performance can facilitate the clinical translation of ultra-high field metabolic imaging in patients with brain diseases. Imaging metabolism brings more specificity to molecular mechanisms of disease in patients, which could help disambiguate the confounding effects^61^ of changes associated with water in anatomical MR imaging, and better guide patient management^41^.

## Methods

Our methodology was developed and implemented on a whole-body 7 T Magnetom MR scanner (Siemens Healthcare, Erlangen, Germany) running the IDEA VB17A software and equiped with the 7 T-SC72CD gradient system capable of 70 mT/m maximum gradient strength and 200 mT/m/s nominal slew rate.

### AC/DC shimming hardware and software

**Figure 9:**
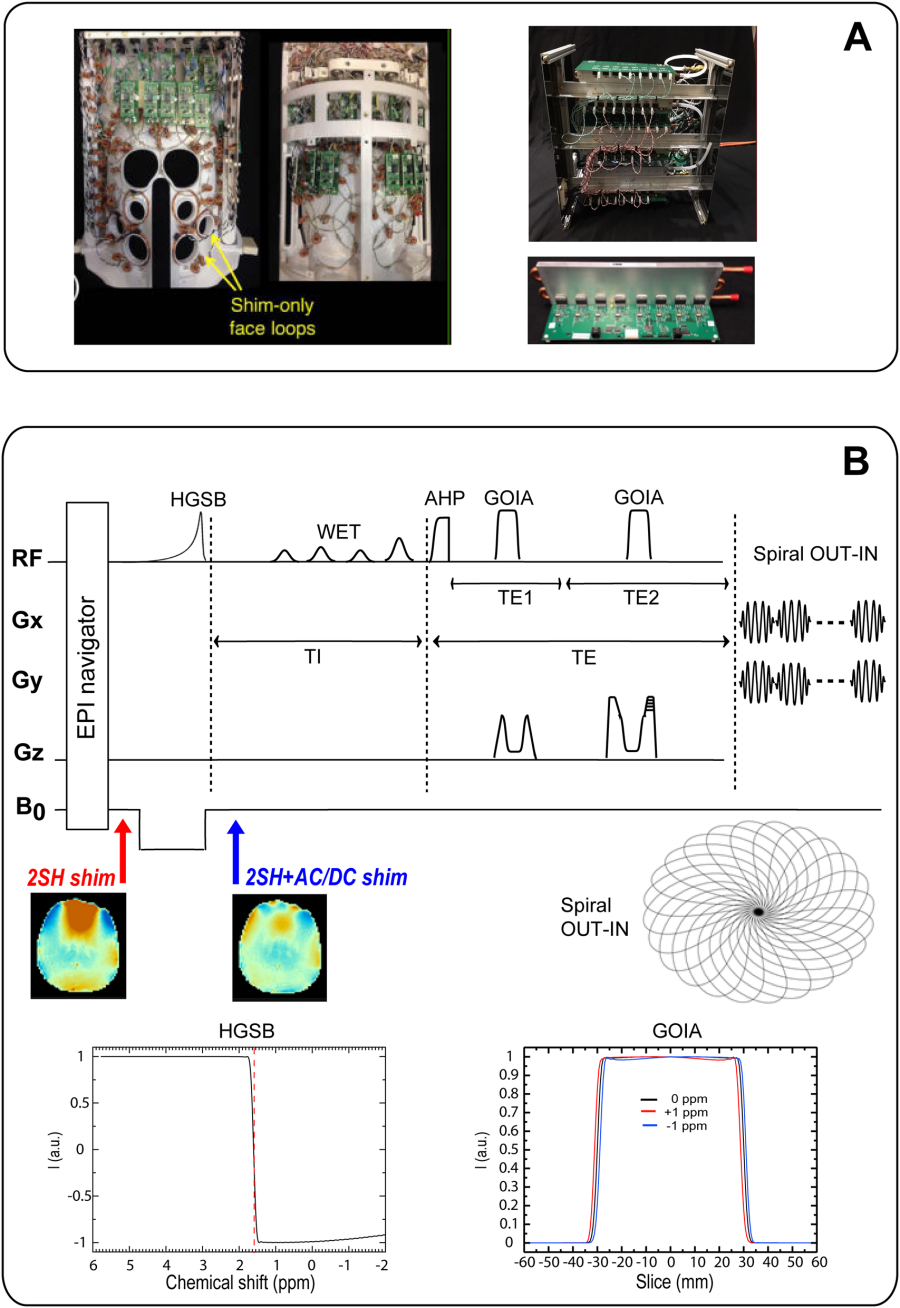
**(A)** Integrated RF-receive/B_0_-shim (AC/DC) array coil (left) and digitally programmable shim supply boards (right). **(B)** 3D MRSI pulse sequence diagram with real-time motion correction and dynamic AC/DC shim update. Echo time TE = 78 ms (TE1/TE2 = 50/28 ms), inversion recovery delay TI = 270 ms, TR = 1,800 ms, FOV = 220 × 220 × 80 mm^3^, matrix 44 × 44 × 8, nominal voxel 5 × 5 × 10 mm^3^, spectral window 2,700 Hz, acquisition time = 11:38 min:s. 2SH = 2nd-order spherical harmonics; AC/DC = *alternating/direct current*; AHP = adiabatic half passage; GOIA = gradient offset independent adiabatic; EPI = echo planar imaging; HGSB = hypergeometric single band; WET = water suppression enhanced through T_1_ effects. Shown at the bottom: B_0_ fieldmaps for 2SH and 2SH + ACDC shimming; the spiral out-in k-space trajectory; the slice profile of GOIA pulses simulated for ± 1 ppm offset; the inversion profile of the HGSB pulse with transition band centered (red dashed line) at 1.6 ppm.

We used recently-developed integrated RF-receive and multi-coil B0 shim coil array hardware for dynamic shimming. The array consists of a 31-channel AC/DC coil array patterned on a close-fitting 3D-printed polycarbonate helmet (3D printer used Fortus 360mc, Stratasys, Rehovot, Israel) (Fig. 9A). The AC/DC coil had 31 RF-receive channels and 31 B_0_-shim channels. In 25 of the loops, B_0_ shim capability was added by creating a DC current path using twisted pair wires and inductive chokes to bridge the DC into the RF circuit. Six of the loops on the inferior-posterior part of the helmet deemed less important for B_0_ shimming were retained as RF receive-only loop. Six four-turn B_0_-shim-only loop coils were placed over the face to target prefrontal cortex building on prior local shim coil work^62^ from other groups. This AC/DC design has been shown in simulations^9^ to provide comparable B_0_ shim performance to a 4^th^-order spherical harmonics shim insert. Thus this hardware setup preserves the receive sensitivity of a close-fitting brain RF receive array, while also providing local B_0_ field control capability using the same array—both of these features are critical for obtaining high-quality MRSI data. A home-built detunable quadrature birdcage coil was used for RF transmit.

Coil loops with a diameter of 9.5 cm made of AWG16 solid wire are arrayed in a hexagonal–pentagonal pattern, with critical overlap to decouple neighboring elements. Other details of the RF coil are given in^26,63^. The AC/DC coil shim currents were driven by a bank of digitally-programmable low-voltage amplifiers that provide DC current up to ± 2.5 A per channel and allow very fast switching in less than 1 ms between different B_0_ field patterns^28^. The output stage devices are mounted to heat sinks with in-laid piping for water cooling. RF excitation was achieved using a detunable quadrature birdcage coil. Dielectric pads were added around the sides of the subject’s head to improve the transmit B_1+_ homogeneity and achieve more uniform MRSI data quality across the whole brain^64^.

The AC/DC shimming was superimposed on the 2SH shimming produced by the scanner hardware. In addition to different hardware combinations, several shimming algorithms were investigated based on the target shim volume, which included only the brain or was a rectangular “box” that included all the head compartments (brain, bone, and scalp) within the MRSI slab. In total five shimming combinations were studied: (1) 2SH_box_, (2) 2SH_brain_, (3) 2SH_box_ + AC/DC_brain_, (4) 2SH_brain_ + AC/DC_brain_, and (5) (2SH_brain_ + AC/DC_brain_)_joint_. The 2SH_box_ is the shimming method provided by the manufacturer to which all users have access. The 2SH_brain_ was previously reported^20^ to improve 2SH_box_, and here we needed to investigate whether AC/DC shimming can provide futher improvement. The joint optimization of 2SH and AC/DC shimming (2SH_brain_ + AC/DC_brain_)_joint_ is expected to make the best use of the orthogonal spatial basis functions of all shimming channels. For calculating the 2SH_box_ shim we employed the vendor supplied software using three shim-iterations with the dual echo steady state (DESS) sequence.

For calibrating the AC/DC shim coils, a vendor-provided two-echo (∆TE = 1.02 ms) gradient echo (GRE) fieldmapping sequence was used to measure the B_0_-fields caused by each individual AC/DC shim coil in a large phantom that completely fills the coil. The same B_0_ mapping sequence was also used to calibrate the scanner 2SH shim coils for the 2SH_brain_ shimming. During the subject measurements B_0_ field maps were measured using the same sequence. All field maps were acquired with field-of-view (FOV) of 224 × 224 × 200 mm^3^, matrix size 112 × 112 × 100, and 2 × 2 × 2 mm^3^ isotropic voxel, acquisition time 1:55 min:s. The phase difference GRE image was spatially unwrapped with FSL PRELUDE^65^, converted to a B_0_-fieldmap, and transfered to an offline computer where the optimal shim currents for the 31 shim channels were computed in under one minute using Matlab’s “quadprog” optimization function (The MathWorks, Natick, MA). A single shim-iteration was performed as follows. Shim currents were calculated using a least squares penalty on ΔB_0_ with the goal of minimizing the standard deviation of the ΔB_0_ over the brain slab of interest that had 70 mm thickness in all subjects. The brain was masked using the FSL Brain Extraction Tool from the magnitude image of the first GRE echo. The objective function is a quadratic program with linear inequality constraints enforcing maximum current per channel (2 A) and total current to the array (25 A). Because of low currents, there is minimal heating of the loops that does not require water cooling of the coil. A similar optimization was performed for 2SH_brain_ using the current limitations and field calibration of the scanner 1st and 2nd order shim coils.

Because the AC/DC shim is optimized over the brain the B_0_ field can vary rapidly outside the brain in the subcutaneous fat, and this can degrade the lipid suppression employed by our sequence. To avoid this problem, a trigger pulse from the sequence is used to switch off the AC/DC shim immediately prior to lipid suppression. A second trigger pulse is used to switch the optimal AC/DC shim back on immediately prior to the water suppression pulses. Because of the low inductance of the coils and rapid switching capability of the control electronics, the fields can be quickly updated during the acquisition in this manner without introducing MRSI artifacts.

In summary, our proposed shim methodology consists of four components: (1) The scanner 2SH-shim, which provides the “baseline” shim, (2) The AC/DC coil, which adds localized ΔB_0_ fields, (3) the possibility to dynamically switch those AC/DC fields within each TR, allowing separate optimization of the shim for metabolite detection and lipid suppression, (4) our shim software, which more readily than the scanner's shim software allows shimming only of the brain instead of the whole head.

### MRSI acquisition and processing

The whole-brain 3D MRSI sequence (Fig. 9B) consisted of five modules: (1) adiabatic spin-echo (ASE) slab excitation^66^ using an AHP4 excitation pulse and two GOIA-W(16,4) refocusing pulses; (2) fat suppression with inversion recovery using an asymmetric adiabatic hypergeometric (HGSB) pulse^67^ and 270 ms inversion time; (3) four-pulse WET module^5^ of 160 ms total duration which was optimized for water suppression at 7 T, and played out within the inversion time; (4) a stack of spiral out-in trajectories^49,50^ designed for the 7 T-SC72CD gradient; (5) volumetric EPI navigator which was interleaved each TR for real-time motion correction, fieldmapping and frequency drift correction^52, 53,68^.

ASE excitation used an AHP4^69^ pulse of 4 ms duration, 5 kHz bandwidth, 12 μT B_1+_ amplitude, and two GOIA-W(16,4)^70^ refocusing pulses of 5 ms duration, 20 kHz bandwidth, 14 μT B_1+_ amplitude (20% above adiabatic threshold). The ASE sequence is a double refocussing sequence similar to PRESS, and the echo time was optimized for 2HG detection at 7 T using TE1/TE2 = 58/20 ms (TE = 78 ms) as proposed for PRESS^45^ sequence. It was verified by simulations that similar to PRESS the ASE with TE1/TE2 = 58/20 ms provides a negative peak for 2HG at 2.25 ppm (Fig. 1). However, the ASE sequence has much smaller chemical shift displacement error (1.5% for 1 ppm at 7 T) compared to PRESS^47^ (21% for 1 ppm at 7 T), and also compensates for B_1+_ transmit inhomogeneity. The slice profile of GOIA pulses used in our MRSI acquisition were simulated for ± 1 ppm chemical shift offset (Fig. 9B), and indicate minimal (2.5%) distortion of the flat top of the passband over 2 ppm chemical shift range. Narrow transition band (10% of the passband) and no out of band exciation are also evident.

The HGSB pulse had 30 ms duration (A = 3.2842; B = 0.1751; C = -1.7; D = 1.4231; Ω = 9.1809), 12 μT B_1+_ amplitude, 2 kHz inversion band, and a transition band of 90 Hz that was centered at 1.6 ppm, providing full inversion below 1.4 ppm and no inversion above 1.8 ppm, hence it suppressed the main lipid peaks at 1.2 and 0.9 ppm while preserving the metabolite SNR. Simulation of the HGSB inversion profile is shown in Fig. 9B. Triggers placed before and after the HGSB pulse were used to switch off/on the AC/DC shimming. WET module used Gauss pulses of 150 Hz bandwidth, flip angles of 83.6°, 99.7°, 74.7°, 160°, and 40 ms interpulse delays. The spiral out-in trajectories were designed for human 7 T MRSI with a spectral window of 2.7 kHz, FOV of 220 × 220 mm^2^, and matrix of 44 × 44, which required a maximum gradient amplitude G_max_ = 14.19 mT/m, and maximum slew rate S_max_ = 158.89 mT/m/s. The spiral out-in design eliminates rewinders used for spiral-out, which increase sampling efficiency and the SNR of MRSI^49,50^.

The following parameters were used to acquire whole-brain slab 3D MRSI: TR = 1,800 ms; TE = 78 ms (TE1/TE2 = 58/20 ms), TI = 270 ms; FOV of 220 × 220 × 80 mm^3^; matrix of 44 × 44 × 8; nominal voxel size 5 × 5 × 10 mm^3^; spectral window 2.7 kHz; 24 angular interleaves, 2 temporal interleaves for spiral out-in; 8 phase encodings; 1 average; acquisition time = 11:38 min:s. The ASE excited a brain slab of 60 mm-thickness that contained six consecutive phase-encoded MRSI slices of 10 mm each. The 60 mm ASE excited slab was slightly smaller than the 70 mm shimmed slab to allow a small margin to transition from inhomogeneous to homogeneous B_0_ field. For all MRSI acquisitions, the specific absorption rate (SAR) was between 60%–95% of the maximum SAR limit as monitored by the MRI system. The acquisition parameters of the navigator were: water selective excitation with flip angle 2°, TR = 8.8 ms, double-echo TE1/TE2 = 3.5/4.5 ms, FOV of 256 × 256 × 176 mm^3^, matrix 32 × 32x22, EPI factor 16, slice partial 6/8 Fourier sampling, bandwidth 4,596 Hz/Px, isotropic voxel 8 × 8 × 8 mm^3^, and acquisition time of 0.6 s as shown in Ref.^68^.

In addition to MRSI metabolite data, water unsuppressed MRSI data (matrix of 22 × 22 × 8; acquisition time of 4:19 min:s) were acquired for coil combination and phasing of metabolite spectra. Coil combination of metabolite data was performed using S/N^2^ weighting^71,72^ of the individual channel data based on the water data. After coil combination, the reconstruction of the non-cartesian sampled data was performed via the nonuniform discrete Fourier transform (NUDFT)^73^, followed by removal of residual lipid signal with the L1 penalty^74^ and spatial Hamming filtering. The raw MRSI data were reconstructed and analyzed with an in-house processing package using MATLAB R2018b, Bash V4.2.25 (Free Software Foundation, Boston, MA, USA), MINC tools V2.0 (McConnell Brain Imaging Center, Montreal, QC, Canada), FSL^75^, and Freesurfer^76^.

MR spectra were phase/frequency corrected and fitted with LCModel^77^ between 1.8 and 4.2 ppm, with a basis-set simulated in GAMMA^78^ using the same pulses and gradient modulation as played by the scanner and including 20 metabolites: D-2-hydroxyglutarate (2HG), aspartate (Asp), creatine (Cr), gamma-aminobutyric acid (GABA), glutamate (Glu), glutamine (Gln), glutathione (GSH), glycine (Gly), glycerophosphocholine (GPC), glycerophosphoethanolamine (GPE), myo-inositol (Ins), lactate (Lac), N-acetyl-aspartate (NAA), N-acetyl-aspartyl glutamate (NAAG), phosphocholine (PCh), phosphocreatine (PCr), phosphorylethanolamine (PE), scyllo-Inositol (Scy), serine (Ser), and taurine (Tau). Total NAA (tNAA) is reported as the sum contribution of NAA and NAAG, total choline (tCho) as the sum of GPC and PCh, and total creatine (tCr) as the sum of Cr and PCr. Note that macromolecule (MM) signals at 2.25 ppm and 4 ppm overlapping 2HG have a T2 relaxation (T2 ~ 20 ms^79,80^) approximately five times shorter than T2 relaxation of 2HG at 7 T (T2 ~ 100 ms, assumed similar to glutamate^81^), hence the MM signals will decay 20 times more than 2HG for TE = 78 ms minimizing the potential quantification bias of 2HG due to MM. Metabolic maps and quality parametric maps for SNR, CRLB and linewidth (FWHM, full width half maximum) are produced for each MRSI data set as estimated by the LCModel fitting routine. Linewidth less than 0.1 ppm and Cramer-Rao lower bound (CRLB) less than 20%, and SNR > 10 were used to determine acceptable goodness of fit for the metabolites quantification.

In addition to MRSI, 3D anatomical imaging was acquired at 1 mm isotropic resolution with T_2_ Fluid Attenuated Inversion Recovery (FLAIR, TR = 5,000 ms, TE = 335 ms, TI = 3,100 ms), and T_1_ multi-echo-MPRAGE (MEMPRAGE^82^, TR = 2,550 ms, TE1/TE2/TE3/TE4 = 1.57/3.35/5.13/6.91 ms, TI = 1,100 ms, flip angle 7°) were also acquired.

### Human subjects

Three healthy subjects (2 females and 1 male, ages 28–33 years) and one mutant IDH1 glioma patient (female, age 42 years) were measured to test our methodology. All experiments and methods were carried out in accordance with relevant guidelines and regulations. The study had ethical approval from the Massachusetts General Hospital Ethics Committee and adhered to the Helsinki Declaration and in accordance to the US government guidelines. Informed consent was obtained from each subject using a study protocol that was approved by the institutional review board (IRB). The mutant IDH glioma patient had previous brain tumor surgery and the *IDH1*-mutational status was established by immunohistochemistry (IHC) analysis using an anti-human R132H antibody (DIANOVA^83^), and also subsequently confirmed by genetic sequencing (SNaPshot^84^).

All five shimming conditions could not be tested in all four subjects because some subjects could not tolerate the entire two hour duration of the full imaging protocol. The 3D MRSI data were measured as following: (a) 2SH_box_ and 2SH_box_ + AC/DC_brain_ in all four subjects; (b) 2SH_box_, 2SH_brain_, 2SH_box_ + AC/DC_brain_, 2SH_brain_ + AC/DC_brain_, (2SH_brain_ + AC/DC_brain_)_joint_ in one healthy volunteer; (c) 2SH_box_, 2SH_brain_, 2SH_box_ + AC/DC_brain_, 2SH_brain_ + AC/DC_brain_ in the patient. We chose to measure the 2SH_box_ and 2SH_box_ + AC/DC_brain_ in all subjects because the comparison of these two shimming conditions is the most relevant: (a) 2SH_box_ is the vendor shimming tool that is used by all users, and (b) 2SH_box_ + AC/DC_brain_ represents the most fair comparison to measure the improvements of AC/DC shimming in addition to the manufacturer shimming. In two subjects (one healthy and one patient) we investigated additional improvements that would be possible when a better shim focused only on the brain (2SH_brain_) could be performed with the scanner hardware. For the glioma patient the tumor contrast-to-noise ratio (CNR) in metabolic images was calculated using the following definition $CNR:=\frac{{mean\left( {\left[ {Metab} \right]_{Tumor} } \right) - mean\left( {\left[ {Metab} \right]_{Background} } \right)}}{{std\left( {\left[ {Metab} \right]_{Background} } \right)}}$, where [Metab] is metabolite concentration in the tumor or outside the tumor (background).

### Phantom measurements

A structural-metabolic phantom (Fig. 2) was custom made with six tubes (25 mm diameter) placed symmetrically inside of a larger cylindrical container (110 mm diameter). The six tubes contained buffered (pH = 7) solution of brain metabolites and 2HG as following: (a) the D-2HG concentration was chosen to be different across the six compartments, respectively 0, 1, 2, 3, 4, and 5 mM, while (b) the same background of brain metabolites was used in all compartments, assuming a tumor metabolic profile with 6 mM of NAA, 8 mM of glutamate, 1 mM of GABA, 4 mM of creatine, 5 mM of choline, 8 mM of myo-inositol, and 4 mM of lactate. The tubes were doped with magnevist 1 ml/l to shorten T_1_, while outside the tubes a buffer aqueous solution without metabolites and magnevist was used to fill the cylinder. All five shim conditions 2SH_box_, 2SH_brain_, 2SH_box_ + AC/DC_brain_, 2SH_brain_ + AC/DC_brain_, (2SH_brain_ + AC/DC_brain_)_joint_ were used to test 3D MRSI metabolic imaging against ground truth.

### Simulations

In order to evaluate the effect of linewidth on 2HG quantification, we performed quantum mechanics simulations (GAMMA^78^) of brain tumor spectra for different spectral linewidths assuming the ASE pulse sequence parameters used in vivo. Synthetic tumor spectra were obtained by combining simulated spectra of 14 brain metabolites and 2HG. The concentration of 2HG was set to 5 mM while for the other 14 metabolites the concentrations were: 4 mM glutamate, 7 mM glutamine, 1 mM GABA, 0.5 mM glutathione, 2 mM glycine, 8 mM myo-ionsitol, 3 mM lactate, 5 mM NAA, 2 mM NAAG, 2 mM PC, 2 mM GPC, 3 mM Cr, 3 mM PCr, 2 mM taurine. T_2_ relaxation times of 132 ms for NAA, 152 ms for phosphocholine and glycerol-phosphocholine, 95 ms for Cr and PCr, and 93 ms for all other metabolites including 2HG were considered^81^.

To mimic the effects of B_0_ inhomogeneity, we applied line broadening in the range of 3–60 Hz (0.01–0.2 ppm at 7 T) with 1 Hz (0.0033 ppm) step size. 10% white noise was added after line broadening in all simulations, yielding SNR of 50 for the narrowest linewidth and SNR of 10 for the widest linewidth. The range of SNR and linewidth in simulations covered the range measured in the in vivo spectra. The simulated spectra were fitted with LCModel^77^ equivalently to experimental spectra.

### Statistical analysis

Statistical analysis was performed using GraphPad Prism (GraphPad Software, Inc. V4.03, CA, USA). Mean differences were compared using the non-parametric Mann–Whitney test with the threshold for statistical significance defined as *P* < 0.05. Data are presented as mean ± standard deviation (SD). Pearson correlation was performed for phantom measurements.

## Supplementary information

Supplementary information
