# Supplementary material for: An integrated RF-receive/B0-shim array coil boosts performance of whole-brain MR spectroscopic imaging at 7 T
Source: Sci Rep. 2020 Sep 14;10:15029. doi: 10.1038/s41598-020-71623-5 (PMC7490394; doi:10.1038/s41598-020-71623-5)
Supplement: Supplementary file 1 — Supplementary information [file 41598_2020_71623_MOESM1_ESM.docx]

**An integrated RF-receive/B_0_-shim array coil boosts performance of whole-brain MR Spectroscopic Imaging at 7 Tesla**

Morteza Esmaeili ^1,2#^, Jason Stockmann ^1#^, Bernhard Strasser ^1#^, Nicolas Arango ^3^, Bijaya Thapa^1^, Zhe Wang ^4^, Andre van der Kouwe ^1^, Jorg Dietrich ^5^, Daniel P. Cahill ^6^, Tracy T. Batchelor ^7^, Jacob White ^3^, Elfar Adalsteinsson ^3^, Lawrence Wald ^1^, Ovidiu C. Andronesi ^1*^

Author Affiliations:

^1^ Athinoula A. Martinos Center for Biomedical Imaging, Department of Radiology, Massachusetts General Hospital, Harvard Medical School, Boston, MA, USA.

^2^ Department of Diagnostic Imaging, Akershus University Hospital, Lørenskog, Norway

^3^ Department of Electrical Engineering and Computer Science, Massachusetts Institute of Technology, Cambridge, MA, USA

^4^ Siemens Medical Solutions, USA, Charlestown, MA, United States

^5^ Division of Neuro-Oncology, Department Neurology, Massachusetts General Hospital, Harvard Medical School, Boston, MA, USA

^6^ Department of Neurosurgery, Massachusetts General Hospital, Harvard Medical School, Boston, MA, USA

^7^ Department Neurology, Brigham’s and Women Hospital, Harvard Medical School, Boston, MA, USA

**
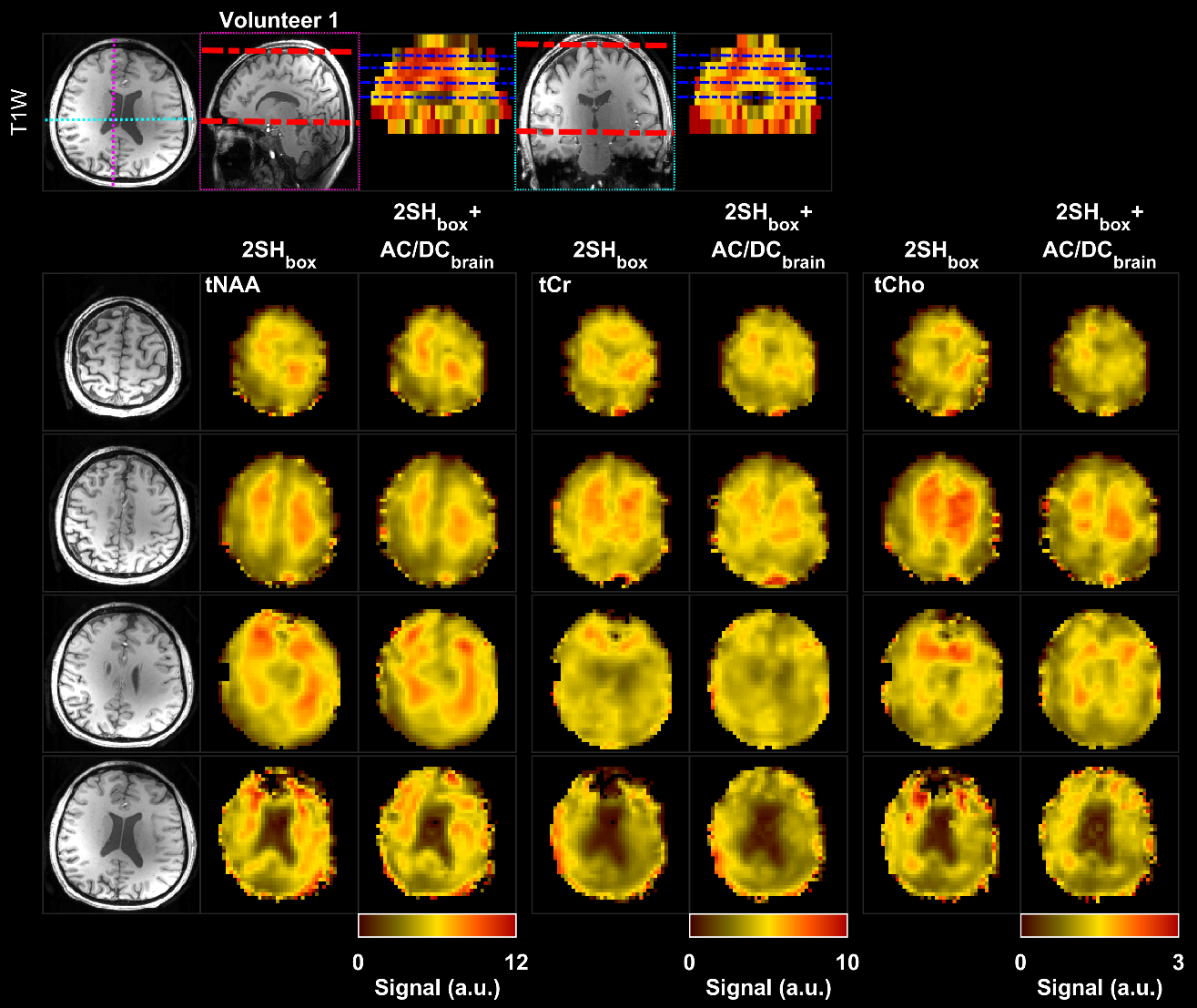
**

**Supplementary Figure 1.** Four slices shown for tNAA, tCho and tCr maps throughout the 3D MRSI slab for the first volunteer acquired with 2SH_box_ and 2SH_box_+AC/DC_brain_ shim conditions. Improvement of the metabolic maps is most evident in the anterior-medial frontal regions for the two most inferior slices which experience the largest B_0_ inhomogeneity with 2SH shimming. The slab position is shown by dashed red lines on the coronal and sagittal MEMPRAGE images at the top. Coronal and sagittal views of the tNAA map obtained with 2SH_box_+AC/DC_brain_ are shown at the top with dashed blue lines indicate the position of the 4 axial slices.

**
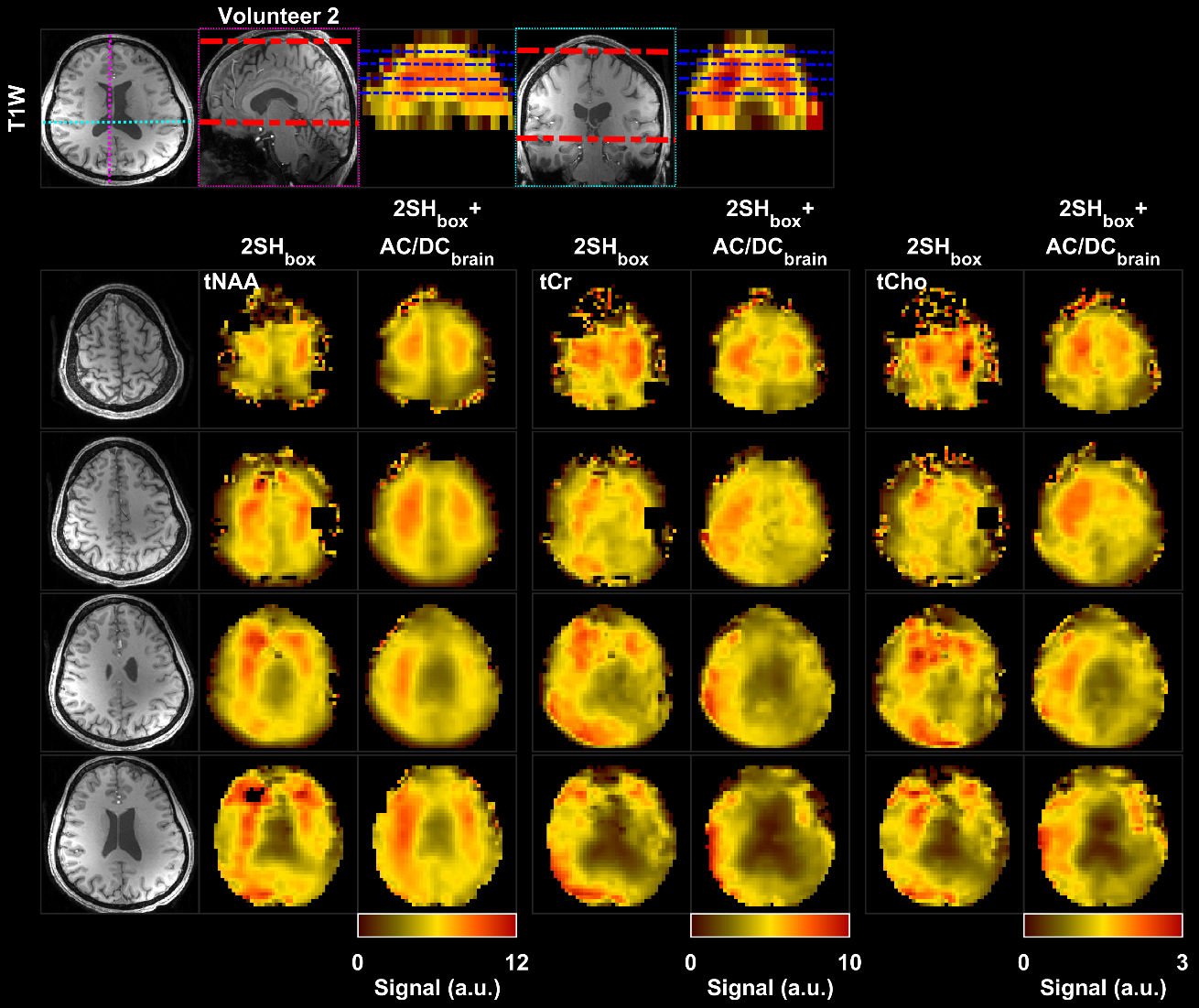
**

**Supplementary Figure 2.**  **Supplementary Figure 1.** Four slices shown for tNAA, tCho and tCr maps throughout the 3D MRSI slab for the first volunteer acquired with 2SH_box_ and 2SH_box_+AC/DC_brain_ shim conditions. Improvement of the metabolic maps is most evident in the anterior-medial frontal regions through all the slices. The slab position is shown by dashed red lines on the coronal and sagittal MEMPRAGE images at the top. Coronal and sagittal views of the tNAA map obtained with 2SH_box_+AC/DC_brain_ are shown at the top with dashed blue lines indicate the position of the 4 axial slices.


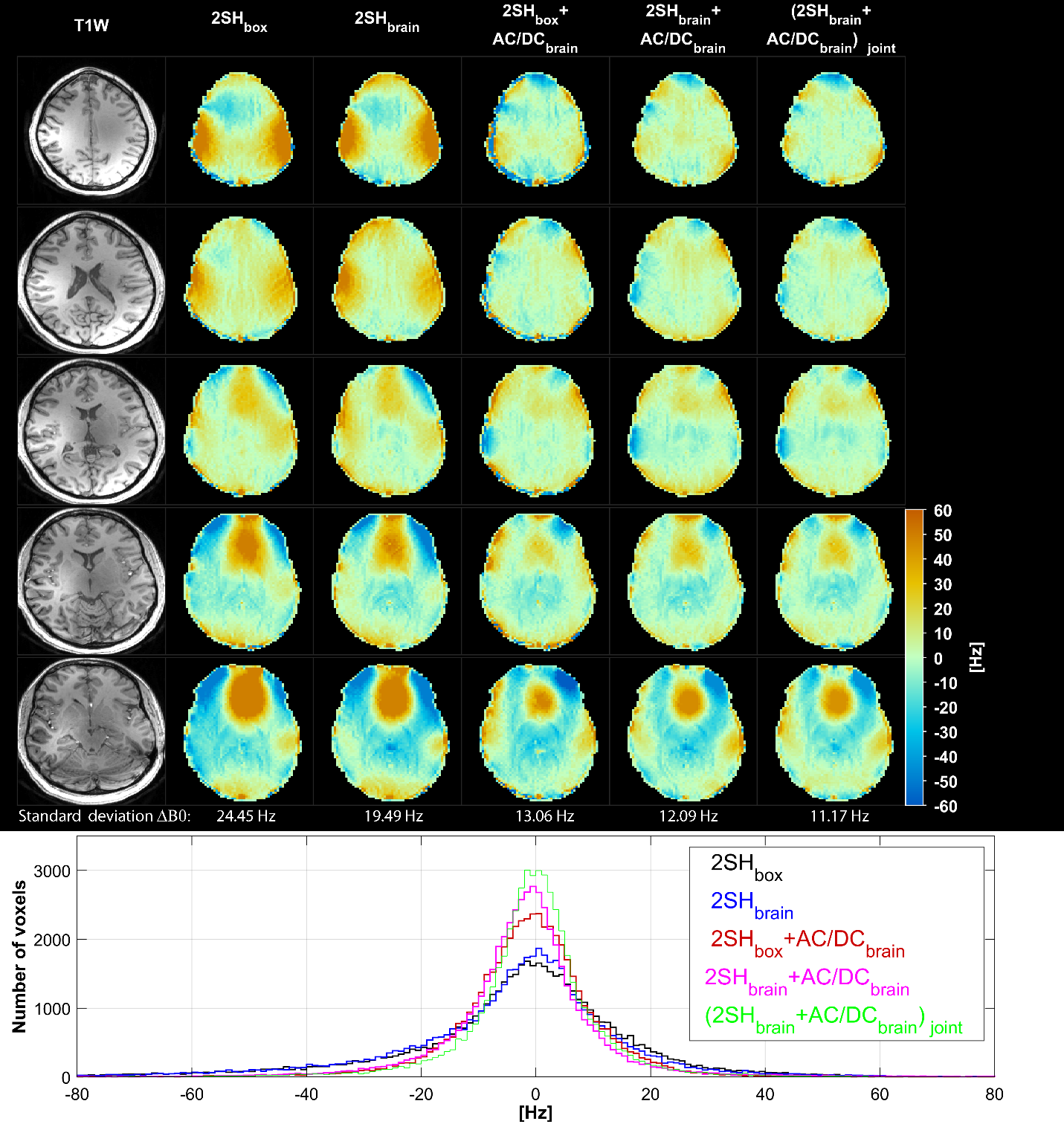


**Supplementary Figure 3.** Comparison of B_0_ fieldmaps in third healthy volunteer under five shimming conditions 2SH_box_, 2SH_brain_, 2SH_box_+AC/DC_brain_, 2SH_brain_+AC/DC_brain_, and (2SH_brain_+AC/DC_brain_)_joint_. Five representative slices are shown through the shimmed whole-brain slab with standard deviation of B_0_ distributions calculated over the entire slab. Histograms of B_0_ distribution over the entire brain slab are shown overlaid at the bottom for all the shimming conditions. MEMPRAGE image is shown in the left-most column.


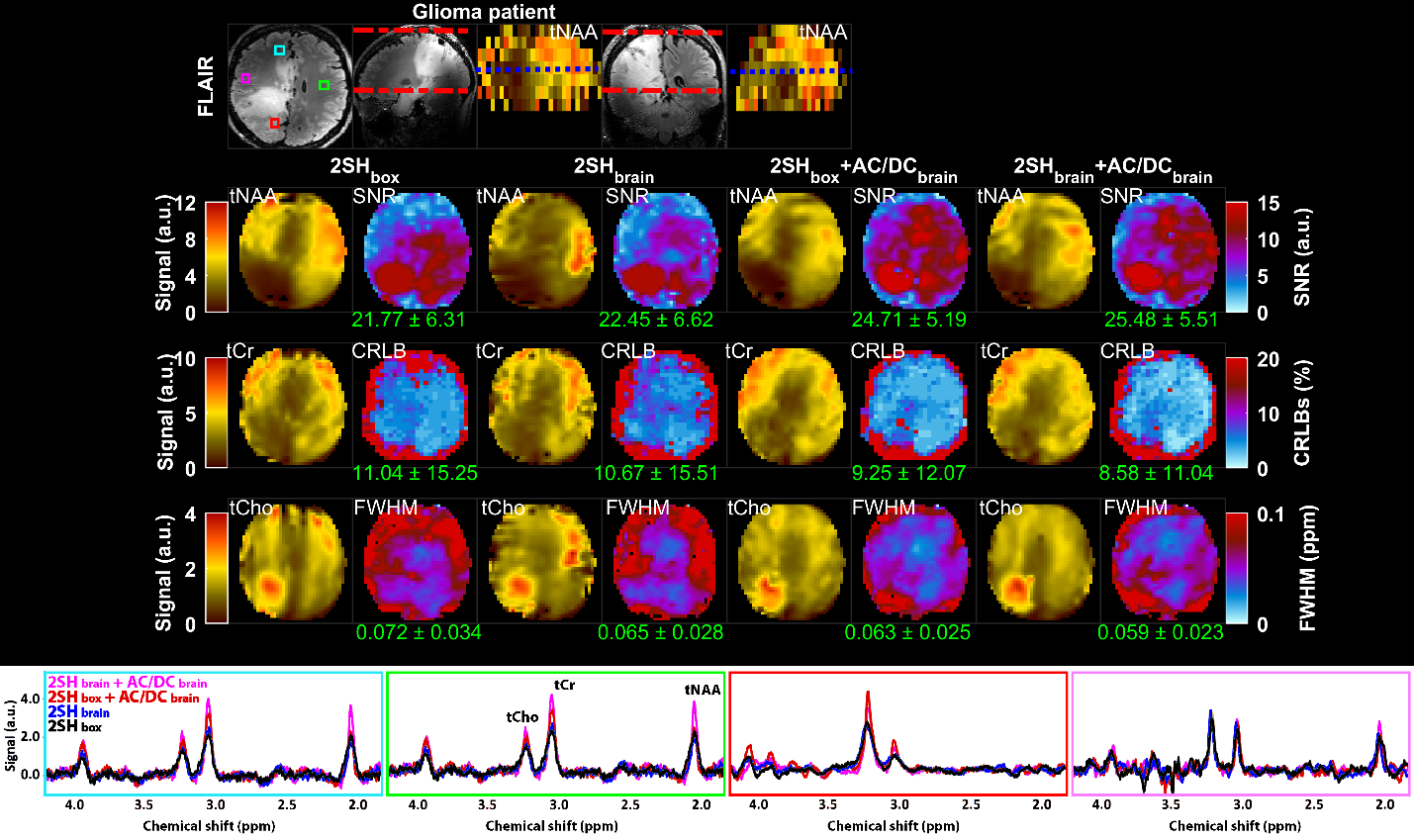


**Supplementary Figure 4.** Comparison of MRSI in the mutant IDH1 glioma patient obtained with the four shimming conditions 2SH_box_, 2SH_brain_, 2SH_box_+AC/DC_brain_, and 2SH_brain_+AC/DC_brain_. An inferior axial slice is shown from the stack of 3D MRSI data as indicated by the blue dashed line in the coronal and sagittal views at the top. The metabolic maps of total NAA (tNAA), total Choline (tCho), total Creatine (tCr), linewidth (FWHM), signal-to-noise ratio (SNR), and Cramer-Rao lower bounds (CRLB of tCr) are shown for all shimming methods. Examples of spectra from frontal (cyan box), left lateral (green box), right lateral (magenta box), and occipital (red box) voxels are shown overlaid for all shimming conditions. The values under the maps indicate the mean and standard deviation calculated over the whole-brain slab. FLAIR anatomical image is shown at the top with dashed red lines indicating the MRSI slab position. Coronal and sagittal views of the tNAA map acquired with 2SH_box_+AC/DC_brain_ are shown next to the corresponding FLAIR images, and the dashed blue line indicates the position of the axial slice.
